# Neural Correlates of Individual Performance Differences in Resolving Perceptual Conflict

**DOI:** 10.1371/journal.pone.0042849

**Published:** 2012-08-20

**Authors:** Franziska Labrenz, Maria Themann, Edmund Wascher, Christian Beste, Bettina Pfleiderer

**Affiliations:** 1 Institute for Cognitive Neuroscience, Biopsychology, Ruhr-University Bochum, Bochum, Germany; 2 IFADO–Leibniz Research Centre, Dortmund, Germany; 3 Department of Clinical Radiology, University of Muenster, Muenster, Germany; CNRS - Université Claude Bernard Lyon 1, France

## Abstract

Attentional mechanisms are a crucial prerequisite to organize behavior. Most situations may be characterized by a ‘competition’ between salient, but irrelevant stimuli and less salient, relevant stimuli. In such situations top-down and bottom-up mechanisms interact with each other. In the present fMRI study, we examined how interindividual differences in resolving situations of perceptual conflict are reflected in brain networks mediating attentional selection. Doing so, we employed a change detection task in which subjects had to detect luminance changes in the presence and absence of competing distractors. The results show that good performers presented increased activation in the orbitofrontal cortex (BA 11), anterior cingulate (BA 25), inferior parietal lobule (BA 40) and visual areas V2 and V3 but decreased activation in BA 39. This suggests that areas mediating top-down attentional control are stronger activated in this group. Increased activity in visual areas reflects distinct neuronal enhancement relating to selective attentional mechanisms in order to solve the perceptual conflict. Opposed to good performers, brain areas activated by poor performers comprised the left inferior parietal lobule (BA 39) and fronto-parietal and visual regions were continuously deactivated, suggesting that poor performers perceive stronger conflict than good performers. Moreover, the suppression of neural activation in visual areas might indicate a strategy of poor performers to inhibit the processing of the irrelevant non-target feature. These results indicate that high sensitivity in perceptual areas and increased attentional control led to less conflict in stimulus processing and consequently to higher performance in competitive attentional selection.

## Introduction

At any time we are only partially aware of the extensive and vivid visual impressions surrounding us. The underlying prerequisites to organize behavior are selective attentional mechanisms that help us to process a small portion of information. The information we pay attention to can be selected either by exogenous or endogenous factors [1]. For example, if we search for a particular object, attention will be focused on the target object and distracting objects in the surrounding will be ignored. Opposed to these top-down mechanisms of attention, very salient objects can also capture attention automatically (bottom-up) [2]–[4]. Previous research examined bottom-up and top-down attentional processes separately, despite several studies stressing that in most situations both processes operate simultaneously [3], [5]. This line of research suggests that bottom-up and top-down processes act in parallel to create a biased representation of the external visual world according to salience and behavioral relevance [6], [7]. More precisely, the detection of a stimulus ‘A’ is determined by (I) the relative saliency of a feature ‘B’ presented in parallel to ‘A’ (i.e., characteristics of the bottom-up channel) and (II) by intentional biases favoring stimulus ‘A’ that are simultaneously adverse for processing feature ‘B’ (i.e., characteristics of the top-down channel). Doing so, top-down biases ‘re-weight’ the relative saliency of stimuli and determine whether feature ‘A’ or ‘B’ is detected [e.g. 3], [5], [8]. Consequently, these feature-based attentional mechanisms can facilitate visual processing by enhancing perceptual representations of previously cued target features and by suppressing representations of competing non-target features [9], [10].

Evidence for this model of attentional selection has repeatedly been referred to in monkey single-cell studies as well as in functional imaging studies. Monkey studies revealed that the inferior temporal cortex (IT) is involved in selecting the objects to which we attend and is crucial for resolving competition [11]–[13]. Likewise, previous studies on humans showed that activity in the visual system is modulated by selective attention, ranging from the thalamic level (corpus geniculatum laterale; CGL) to striate (V1) and extrastriate (V2, V3, V4, MT/MST) visual areas [14]–[16]. Top-down influences that induce biasing effects in the above mentioned areas are mediated via a fronto-parietal network, including the frontal eye field (FEF), superior parietal lobule (SPL) as well as intraparietal sulcus [17]–[19]. Besides the identification of target projection sites of attentional processes, functional imaging studies indicated that top-down attentional processes are distinct neural processes where cortical responses are either enhanced or suppressed. This is comparable to the concept that task-relevant stimuli elicit an increase in cortical activation whereas a decrease in cortical activation is related to task-irrelevant stimuli [13], [20]–[21]. This concept has been corroborated for various areas of the visual cortex like areas V2, V4, the middle temporal (MT) and medial superior temporal (MST) areas, where sensory suppression takes place when stimuli compete with each other [13], [16], [22]–[25].

Within the framework of selective visual attention the role of stimulus saliency (bottom-up channel characteristics) in determining the outcome of attentional selection processes has been subject to several studies, showing that stimuli irrelevant for a given task may nevertheless capture attention when they are sufficiently salient [26]–[28]. To investigate the interaction of bottom-up and top-down attentional processes and the concomitant bias on visual selection we employed a change detection task, similar to the one used by Wascher and Beste [29]. The subject's task was to detect changes of luminance and to ignore changes of orientation during trials in which the stimulus dimensions could either change singularly or simultaneously and spatially separated or joint. If both luminance and orientation change simultaneously but spatially separated, a perceptual conflict is induced in which subjects have to enhance the processing of the less salient but task-relevant luminance change against the competing and more salient orientation change. In a recent study, Wascher and Beste [29] suggested that saliency of the distracting stimuli is predictive for the portion of detected relevant targets when both relevant and irrelevant information are spatially separated. However, while this study demonstrated a cascade of subsequently occurring attentional allocation and re-allocation processes to distractor and target stimuli using ERPs [29], they were not able to examine which brain areas and brain networks underlie mechanisms of perceptual competition as operationalized by their paradigm. Thus, it remains unclear what brain areas mainly determine performance in situations of competitive attentional selection. In other words: What brain areas are differentially activated in “good” compared to “poor performance” in situations of competitive attentional selection?

The investigation of individual differences has taken on greater significance in cognitive research and provides a powerful approach for elaborating the functional role of distinct brain regions [30]. During tasks that focus on selective attention, subjects encounter different capacity and ability constraints and thus inevitably perform differently. By identifying correlations between task performance and brain activity this interindividual variability can be explained in terms of strategic differences that are reflected in neural efficiency. Therefore, brain areas will be revealed that are necessary to perform a certain task as well as brain areas that are indicative of deficient and superior task performance.

Based on the notion that perceptual performance in the given task depends on the sensitivity for relevant features, we hypothesized that good performers will present an enhanced BOLD response in occipito-temporal areas encompassing the extrastriate cortex (BA's 18, 19) during trials, where a perceptual conflict between different stimuli is evident. Moreover, in good performers enhanced BOLD responses should be evident in fronto-parietal networks as expression of intensified top-down biasing processes resolving the perceptual conflict between the stimuli. In contrast, poor performers should most likely be influenced more by distracting stimuli which are irrelevant for the task, than by the less salient but relevant stimuli. Poor performers should therefore show a less active top-down network in comparison to good performers.

## Materials and Methods

### Participants

Twenty-four healthy individuals (11 females) participated in the fMRI study (mean age±SD, 23.33±2.53 years; range 20–30 years). All participants were right-handed (mean 90.29, SD = 10.04) based on Edinburgh Handedness Inventory [31], reported no history of neurological or psychiatric disorders and had normal or corrected to normal vision. The study was approved by the Medical Council of Westfalen-Lippe in compliance with the Code of Ethics of the World Medical Association (Declaration of Helsinki). Every subject gave written informed consent and was either compensated for participation in the study with money (€20) or course credits.

### Stimuli and experimental procedure

**Figure 1:**
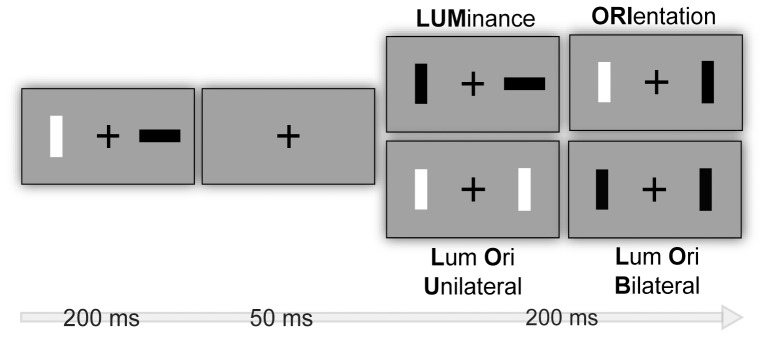
Stimuli set-up and experimental procedure. Within-subject factor type of stimulus change comprises luminance change of one bar (LUM), orientation change of one bar (ORI), luminance and orientation change of one bar (Luminance Orientation Unilateral = LOU) and luminance and orientation change across two bars (Luminance Orientation Bilateral = LOB). Participants were instructed to detect a change in luminance in a sequence of two frames.

The task applied is similar to the one used by Wascher and Beste [29] and depicted in Figure 1. The stimulus material consisted of two rectangular bars which were oriented either horizontally or vertically, presented 2° visual angle left and right from a fixation cross and were either brighter or darker than the background (30 cd/m^2^) with a Fechner-Contrast of ±0.2.

Each trial consisted of the successive presentation of two frames. The first frame was presented for 200 ms, followed by an interstimulus interval of 50 ms, in which only the fixation cross was visible to mask the transient related to the change. Afterward, the second frame was presented for 200 ms. In order to manipulate bottom-up attentional bias luminance and orientation changed randomly between these two frames constituting four conditions: If only brightness of a stimulus changed between first and second frame, a transient of luminance occurred which will be further referred to as condition LUM. If only orientation changed, the impression of an apparent motion was given (ORI). If luminance and orientation changed at the same spatial location, both transients coincided (luminance and orientation unilateral = LOU). If luminance and orientation changed simultaneously but spatially distributed (luminance and orientation bilateral = LOB) a perceptual conflict was induced because subjects were instructed to detect the less salient luminance transient and to ignore the more salient orientation transient. To indicate the luminance change subjects responded by pressing the left button on a touch pad if the luminance change appeared on the left side and the right button if the luminance change appeared on the right side. Trials in which only orientation changed were no-go trials and subjects had to refrain from responding. Stimulus combinations were randomly intermixed and in consecutive trials the different conditions alternated in a way that no condition was presented twice in succession. Overall 640 trials were presented, 160 for each condition.

### Data analysis

#### Behavioral data analysis

Responses were recorded from the onset of the second frame. Response times (RTs) were defined as the time between stimulus change in the second frame and button press. Error categories comprised response errors (i.e., false button presses and false alarms in the no-go condition when only orientation changed) and misses (i.e., no response was registered within 1.500 ms after stimulus onset). These behavioral parameters were then analyzed across the total sample and according to the extreme group's approach [32]. As the measurement of selective attention yields continuous scores it might be difficult to interpret the relevance of behavioral differences and the underlying neural substrates when considering the entire sample. But even if subjects are assigned to continuous subgroups based on the median score of some variable of interest, these groups may not reveal both ends of the continuum that underlie individual differences because the differentiation between good and poor performance may be blurred. Therefore, another design to maximize the power to detect individual differences and to further reduce the variance between performance subgroups relative to the variance within the entire sample is the assignment of subjects to extreme groups covering only lowest and highest scores of the variable of interest. To assess the impact of individual performance differences, we calculated a score to indicate the subject's susceptibility to distractor interference. Therefore, errors in trials in which the target stimulus was presented solely (LUM) were subtracted from errors of trials presenting a competing distractor (LOB) (LOB minus LUM). In this sense, we can provide a quantitative value to determine the subject's susceptibility to distraction and to investigate the strength of top-down attentional control allocated to the task-relevant stimulus in the absence and presence of the competing distractor by means of a relativization to the basic error rate. This performance score was then used as variable of interest to divide the total sample into extreme groups according to both extremes of the distribution curve. Thus, subjects performing 33% below the mean performance score (<11.04% errors) and 66% above mean performance score (>16.67% errors) were indicative of good and poor performers, respectively.

RTs and error rates were analyzed for all subjects by means of a repeated-measures ANOVA using within-subject factor “trial type” defining the type of stimulus change (LUM, ORI, LOU, and LOB) and for analyzing individual performance differences additionally with between-subject factor “performance” defining good and poor performance on the task. Significances were Greenhouse-Geisser corrected and additional post-hoc tests were performed and Bonferroni-corrected if necessary. All statistical tests were performed with Predictive Analytics Software (PASW) V 18.0.

#### fMRI data analysis

Functional MR imaging was carried out with a 3T Philips Scanner (Gyroscan; Philips, Best, Netherlands) with a standard 8-channel receiver head coil. Stimuli were presented on a monitor projecting to a mirror positioned on the head coil. Before the fMRI datasets, T1-weighted images were acquired with time of repetition TR = 3500 ms, time of echo TE = 35 ms, flip angle 90°, matrix dimensions 256×256 mm, a field of view FOV = 210 mm and 36 oblique slices. Image processing and statistical analysis of the fMRI images were done by SPM5 standard routines and templates (Wellcome Department of Cognitive Neurology, London, U.K.). Pre-processing steps involved realignment, normalization (resulting voxel size 2 2 2 mm^3^) and smoothing (8-mm isotropic Gaussian kernel). Data were filtered with a high-pass filter applying a cut-off period of 128 sec. Motion parameters of the realignment step during pre-processing were integrated into the model to control for regressive effects by motion. Events of interest were time-locked to the onset of the first frame. After pre-processing the fMRI data, individual data analysis was performed. Both correct and incorrect trials (i.e., false button presses and false alarms in the no-go condition) were entered into analysis separately. As subjects were assigned to good and poor performers on basis of their performance, the number of successful and error trials and consequently the percentage of trials in proximity to an error differed considerably. However, it has been shown that brain activation remains consistent across trials. Thus, the obtained activation patterns of certain brain regions are highly indicative of their functional relevance for cognitive mechanisms although only a small number of trials can be used for analysis [33].

For each subject four BOLD contrast differences (t-contrasts) were determined as a function of BOLD signal change compared to noise level by modeling the corresponding regressors (LUM, ORI, LOU, LOB) to obtain scaled beta weights for each conditional event [34]. These conditions of interest were convolved with the canonical hemodynamic response function (HRF) with no derivatives and were entered into second-level analysis using the general linear model (GLM). Anatomical localization of activated brain regions was determined by reference to standard stereotaxic atlas by Talairach and Tournoux [35].

To gain valuable insights into the relationship between individual differences in task performance and brain activation we used an experimental task that aims at producing highly varying performance across subjects. Moreover, we adopted an integrative approach by modeling associations between task performance and brain activation both within and between subjects. This approach comprises random effects analyses across the total sample and performance groups, regression analyses, ANOVAs and the calculation of beta weight estimates.

Random effect analyses were performed on the individual subject's contrast images to obtain group contrast maps for conflict trials (LOB) across the entire sample and extreme groups. To test hypotheses regarding the region dependence in performance-brain activation relationships multiple regression analyses across the whole brain were conducted using performance scores as regressors. Although the use of reaction time measures as a predictor of brain activation seems to be preferable this approach has been shown to be affected by temporal BOLD summation and consequently increases the probability of type I errors [36].

To compare signal activations between good and poor performers a full factorial ANOVA was applied on both successful and error trials with “conflict trial” as factor with two levels comprising good and poor performers. For all reported analyses a Monte Carlo simulation of the brain volume was conducted to establish an appropriate voxel contiguity threshold [37]. Assuming an individual voxel type I error of p<.005, a cluster extent of 70 contiguous resampled voxels was indicated as sufficient to correct for multiple voxel comparisons at p<.005. Thus, voxels with an uncorrected significance level of p<.005 with a minimum cluster size of at least 70 voxels were reported for all analyzes.

Derived from previous neuroimaging studies [17]–[19] we have chosen three sets of regions encompassing frontal, parietal and visual regions that should capture the main contributions of bottom-up and top-down attentional control. Specific coordinates of brain regions could not be used because the task applied in this study has been developed only recently. Therefore, regions of interest (ROIs) were defined as the activation clusters obtained from regression analyses and ANOVAs. Conforming to the center coordinates of the ROIs (Table 1) indicated by results of the regression analyses and ANOVAs, contralateral ROIs were acquired additionally to consider lateralization effects. Beta weights were derived from all reported ROIs and were calculated by means of beta-weighted images for each region of interest (ROI) and compared using ANOVAs with subsequent Bonferroni-corrected pair-wise comparisons. Finally, beta weights were entered into a linear regression model to verify the association between brain activation and task performance.

**Table 1:** ROIs used for calculation of activation beta estimates.

Region	Talairach coordinates		
	x	y	z
SUCCESSFUL TRIALS			
ROIs derived from regression analyses			
L Inferior frontal gyrus (BA 9)	−50	20	20
R IFG (BA 9)	50	20	20
L Angular gyrus (BA 39)	−52	−62	30
R AG (BA 39)	52	−62	30
L Middle temporal gyrus (BA 21)	−48	0	−16
R MTG (BA 21)	48	0	−16
L Superior temporal gyrus (BA 22)	−48	−2	−4
R STG (BA 22)	48	−2	−4
L Cingulate Gyrus (BA 24)	−6	18	26
R CG (BA 24)	6	18	26
L Cingulate gyrus (BA 32)	−14	16	26
R CG (BA 32)	14	16	26
L Anterior cingulate (BA 33)	−2	18	18
R ACC (BA 33)	2	18	18
ROIs derived from ANOVA			
L Rectal gyrus (BA11)	−4	10	−22
R RG (BA11)	4	10	22
L Cuneus (BA 18)	−18	−74	18
R Cun (BA 18)	18	−74	18
L Lingual gyrus (BA 18)	−4	−66	2
R LG (BA 18)	4	−66	2
L Precuneus (BA 19)	−34	−66	36
R Precuneus (BA 19)	34	−66	36
L Anterior cingulate (BA 25)	0	14	−8
L Middle temporal gyrus (BA 39)	−32	60	28
R MTG (BA 39)	32	−60	28
L Inferior parietal lobule (BA 39)	−50	−62	38
R IPL (BA 39)	50	−62	38
L Inferior parietal lobule (BA 40)	−38	−50	34
R IPL (BA 40)	38	−50	34
ERROR TRIALS			
ROIs derived from regression analyses			
L Medial frontal gyrus (BA 6)	−14	−24	58
R MFG (BA 6)	14	−24	58
L Superior frontal gyrus (BA 8)	−14	46	42
R SFG (BA 8)	14	46	42
L parahippocampal gyrus (BA 28)	−18	−18	−26
R PHG (BA 28)	18	−18	−26
L Superior temporal gyrus (BA 39)	−48	−56	24
R Superior temporal gyrus (BA 39)	48	−56	24

## Results

### Behavioral data

**Figure 2:**
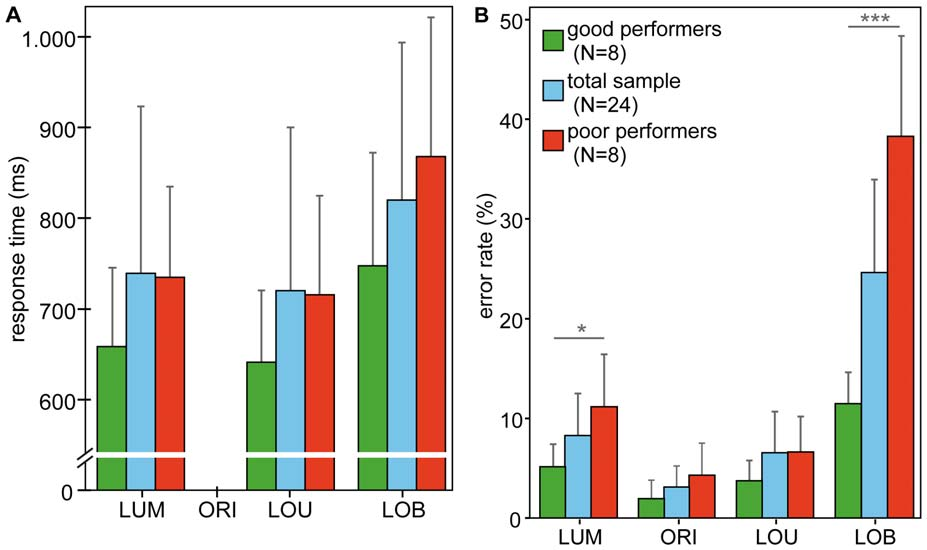
Behavioral results. Mean response times (**A**) and error rates (**B**) for the total sample (N = 24; shown in blue), good performers (N = 8; shown in green) and poor performers (N = 8; shown in red); error bars depict standard error. Both response times and error rates were highest on perceptual conflict trials (LOB) in comparison to non-conflict trials (LUM, ORI, LOU). Missing reaction times for the ORI condition indicate no-go trials subjects were instructed not to respond to.

Figure 2 illustrates the distribution of mean response times (panel A) and mean error rates (panel B) for trial types. Corresponding descriptive statistics are listed in Table 2. Response times were negatively affected by the presence of a competing distractor (LOB trials) for both, the total sample and the performance subgroups as indicated by a main effect of trial type. This effect progressively increased in effect size from total sample (F_(2,48)_ = 47.759, η^2^>.67, p<.001) to the extreme groups (F_(2,28)_ = 42.197, η^2^>.75, p<.001) indicating that overall response times were lowest on LOU trials and highest on LOB trials and the difference between LUM and LOU trials being larger for extreme groups (p = .004) than for the total sample (p = .008). Moreover, the separate analysis of the extreme performance groups did not yield a significant difference between good and poor performers (F_(1,14)_ = 2.144, p = .165) implying that better performance cannot be explained by higher response times.

**Table 2:** Behavioral data.

	Total sample	Good performers	Poor performers
	(N = 24)	(N = 8)	(N = 8)
RT (ms) LUM	720.41±147.44	658.86±103.38	734.46±119.88
RT (ms) LOU	706.12±147.52	641.33±94.68	715.79±130.19
RT (ms) LOB	817.96±181.55	747.18±149.32	867.94±183.67
ER (%) LUM	10.40±6.67	5.16±2.71	11.17±6.91
ER (%) ORI	4.77±6.32	1.95±2.20	4.30±3.86
ER (%) LOU	8.65±7.50	3.75±2.43	6.64±4.25
ER (%) LOB	26.35±13.96	11.48±3.76	38.28±12.06

Response times and error rates across all trial types for both the total sample and performance groups.

Similarly, for error rates a highly significant main effect of trial type has been revealed across the total sample (F_(3,72)_ = 36.126, η^2^ = .60, p<.001) and extreme groups (F_(3,42)_ = 52.424, η^2^ = .79, p<.001) indicating that detection of the task-relevant luminance change was highly impaired on perceptual conflict (LOB) trials. Furthermore, the separate analysis of the extreme groups yielded a significant interaction between trial type and performance (F_(3,42)_ = 24.554, η^2^ = .64, p<.001) indicating that a difference between good and poor performers was evident on perceptual conflict (LOB) trials (t_(14)_ = −3.935, p = .001) but not on other trial types (all t′s_(14)_<.583, all p′s>.569).

### fMRI data

#### Random effects analyses at total sample and extreme performance levels

**Figure 3:**
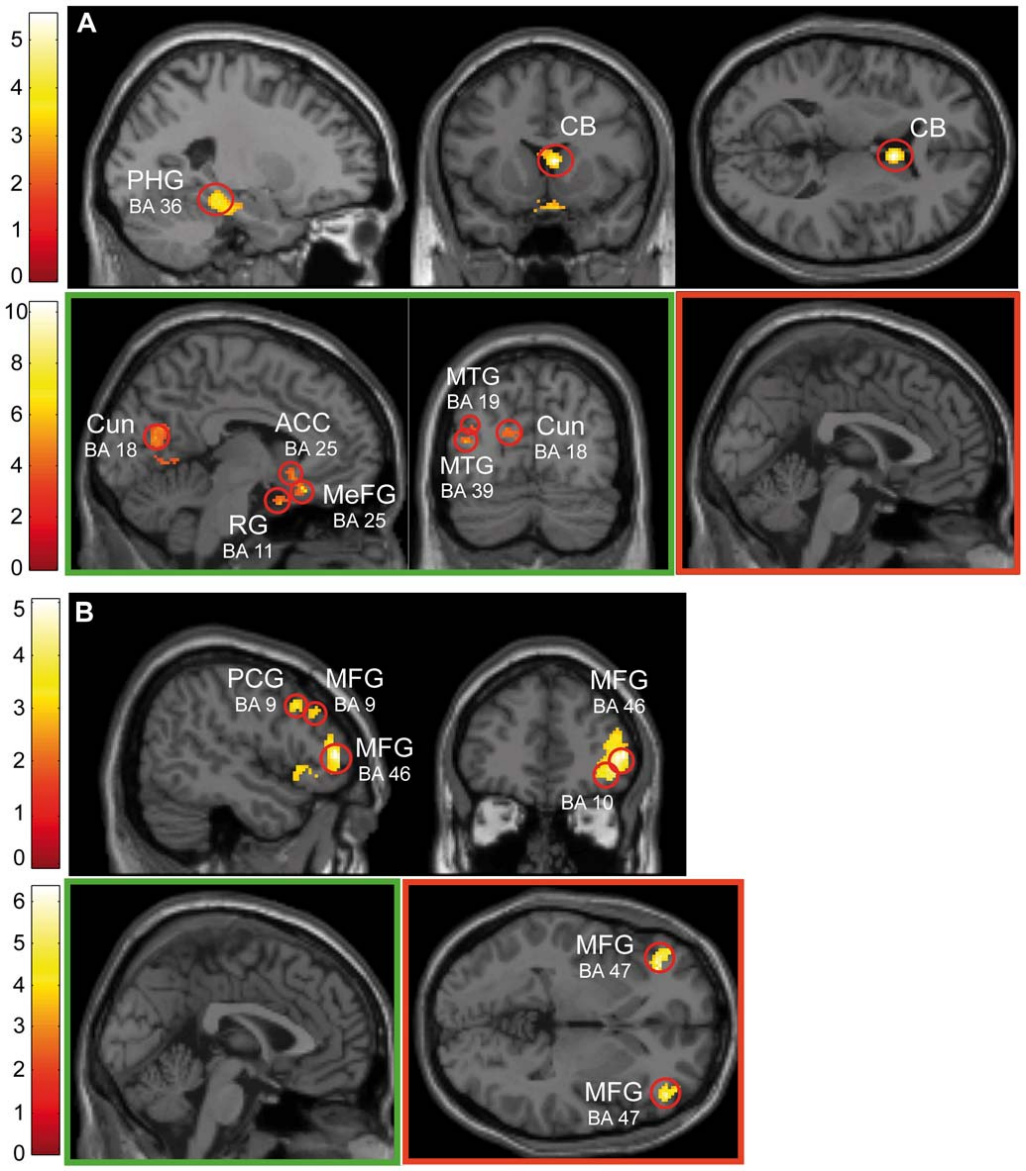
Distribution of brain activation across successful and error trials during perceptual conflict. Random effects analyses of perceptual conflict trials (p<.005 uncorr., k>70 voxels) showed different activation patterns across the total sample and performance groups. (**A**) On successful trials across the total sample (upper panel) an activation pattern emerged encompassing PHG (BA's 35, 36) and caudate body. Good performers (lower left panel; shown in green) exhibited a more widely distributed activation pattern including fronto-limbic (BA's 11, 25), temporal (BA's 19, 39) and more posterior regions in the posterior cingulate (BA 30) and PHG (BA's 28, 35). Poor performers (lower right panel; shown in red) revealed no significant activation. (**B**) On error trials the total sample (upper panel) showed activation in fronto-parietal regions encompassing BA's 6, 9, 11, 32, 46 and 40. While for good performers (lower left panel; shown in green) no significant activation was seen poor performers (lower right panel; shown in red) revealed activation clusters in IFG and MFG (BA's 46, 47).

The results of the random effect analyses on successful and error trials across the total sample (N = 24) and performance subgroups (N = 8 each) are depicted in Figure 3 (see Table 3 for voxel locations and corresponding statistical information). We have included only perceptual conflict (LOB) trials in which changes of luminance and orientation occurred simultaneously but spatially separated to emphasize the interaction between bottom-up and top-down attention and likewise because performance differences on the behavioral level were exclusively evident on these trials.

**Table 3:** Random effects analyses on successful trials.

Region	Talairach coordinates			Z	k_E_
	x	y	z		
**Total sample (N = 24)**					
L Parahippocampal gyrus (BA 36)	−24	−30	−16	3.83	754
L Culmen	−16	−32	−14	4.23	
	−8	−36	−8	3.73	
R Caudate body	6	12	10	4.80	127
**Good performers (N = 8)**					
L Rectal gyrus (BA 11)	−2	8	−26	3.67	70
L Medial frontal gyrus (BA 25)	−6	26	−18	3.70	88
L Anterior cingulate (BA 25)	−4	18	−8	3.35	
L Middle temporal gyrus (BA 19)	−34	−80	24	3.56	95
L Middle temporal gyrus (BA 39)	−42	−74	14	3.25	
L Cuneus (BA 18)	−10	−68	14	3.63	797
R Posterior cingulate (BA 30)	10	−56	10	3.87	
R Culmen	6	−60	−4	4.30	
L Parahippocampal gyrus (BA 28)	−18	−14	−24	3.50	149
L Parahippocampal gyrus (BA 35)	−28	−26	−20	2.78	
L Culmen	−24	−32	−24	3.59	

Brain regions showing significant activations across the total sample (N = 24) and good performers (N = 8) on successful perceptual conflict trials (p<.005 uncorr., k>70 voxels). No activation clusters were evident in poor performers (N = 8).

On successful trials (Figure 3A; Table 3) the total sample (LOB) (upper panel) elicited activation in the parahippocampal gyrus (BA's 35, 36) and caudate body. By comparison, the subgroup of good performers (Figure 3A, lower left panel; Table 3) exhibited additional and more widely distributed brain activation in fronto-limbic (BA's 11, 25, 28, 30, 35), temporal (BA's 19, 39) and occipital (BA 18) areas. In contrast, poor performers (Figure 3A, lower right panel) exhibited no activation clusters within the same threshold on successful trials.

On error trials (false button presses and misses) (Figure 3B; Table 4) activation of a fronto-parietal network emerged, encompassing BA's 6, 9, 11, 32, 46, inferior parietal lobule (BA 40) and the claustrum (upper panel). Unlike successful trials, for good performers (Figure 3B, lower left panel) no activation clusters reached significance on error trials whereas the activation pattern obtained from poor performers (Figure 3B, lower right panel; Table 4) was evident in the prefrontal cortex (BA's 46, 47).

**Table 4:** Random effects analyses on error trials.

Region	Talairach coordinates			Z	k_E_
	x	y	z		
Total sample (N = 24)					
R Middle frontal gyrus (BA 6)	26	2	38	3.31	162
R Cingulate gyrus (BA 32)	22	14	26	3.20	
R Claustrum	26	18	14	3.34	
R Precentral gyrus (BA 9)	46	18	34	3.27	121
R Middle frontal gyrus (BA 9)	36	20	34	3.08	
R Middle frontal gyrus (BA 9)	42	34	36	3.31	78
	50	30	30	2.72	
R Sub-gyral (BA 10)	42	40	0	3.95	815
R Middle frontal gyrus (BA 11)	26	38	−8	3.52	
R Middle frontal gyrus (BA 46)	50	44	6	4.12	
L Inferior parietal lobule (BA 40)	−46	−48	58	3.55	208
	−58	−38	36	3.11	
	−58	−40	48	3.00	
Poor performers (N = 8)					
L Middle frontal gyrus (BA 46)	−48	44	8	3.42	182
L Middle frontal gyrus (BA 47)	−34	36	−2	3.55	
	−42	36	−4	3.43	
R Middle frontal gyrus (BA 46)	50	44	8	2.86	121
R Inferior frontal gyrus	48	42	0	3.37	

Brain regions showing significant activations across the total sample (N = 24) and good performers (N = 8) on erroneous perceptual conflict trials (p<.005 uncorr., k>70 voxels). No activation clusters were evident in good performers (N = 8).

#### Correlative links between task performance and brain activation

**Figure 4:**
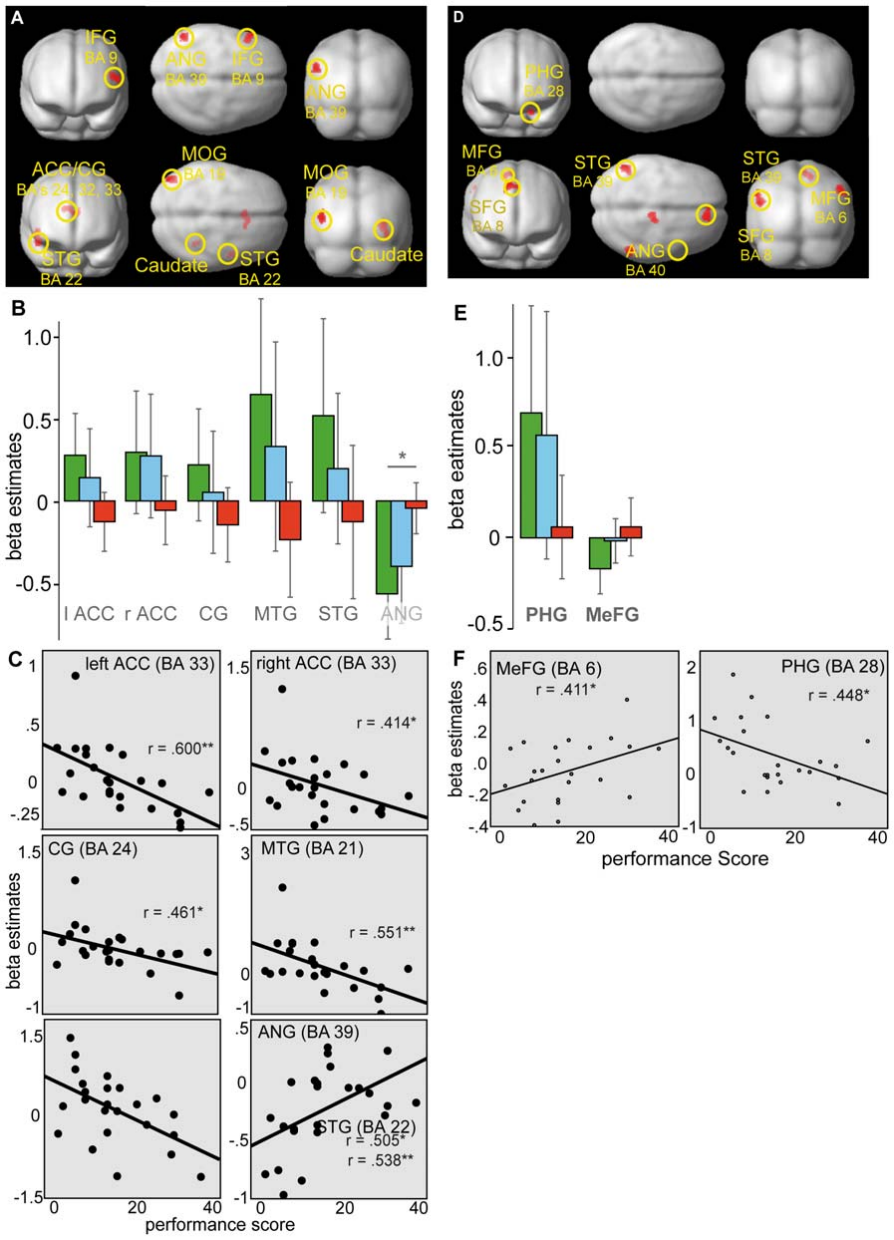
Regression analyses during perceptual conflict trials. (**A**) On successful perceptual conflict trials regression analyses revealed a significant positive correlation between task performance and brain activations in IFG (BA 9) and ANG (BA 39) (upper panel). A negative correlation (lower panel) was evident in ACC (BA 33), CG (BA's 24, 32), middle (BA 21) and superior temporal gyrus (BA 22), PHG (BA 19), MOG (BA 19) and caudate tail. (**B**) Activation clusters derived from the regression analyses across successful trials revealed significant differences in beta estimates between good and poor performance only in the ANG (BA 39) by means of an independent samples t-test. (**C**) However, regression analyses showed significant positive and negative correlations in the left ANG (BA 39), bilateral ACC (BA 33), left CG (BA 24) and right MTG (BA 21). Scatter plots show regional mean beta estimates for each subject plotted against their performance scores. (**D**) On error trials task performance led to a robust positively correlated activation in the PHG (BA 28) (upper panel). The negatively correlated performance-brain relationship indicated activations in the medial (BA 6) and superior frontal gyrus (BA 8), STG (BA 39) and BA 40 encompassing the IPL and ANG (lower panel). (**E**) Activation clusters derived from the regression analyses across error trials revealed no significant differences in beta estimates between good and poor performance. (**F**) As indicated by regression analyses a negative correlation yielded significance in the PHG (BA 28) and a significant positive correlation in the MeFG (BA 6). Scatter plots show regional mean beta estimates for each subject plotted against their performance score.

As we aimed at linking individual performance differences to human brain functioning on perceptual conflict trials, we performed regression analyses across both successful and error trials to investigate the impact of task performance on strategic differences and efficiency of neural processes. The results of the regression analyses using subjects' error rates and regional beta weight estimates on perceptual conflict trials was characterized by both positive and negative performance-brain activation relationships in several cortical and subcortical brain regions (Figure 4 and Table 5).

**Table 5:** Regression analyses on successful and erroneous trials.

Region	Talairach coordinates			Z	k_E_
	x	y	z		
**SUCCESSFUL TRIALS**					
**Positive correlation**					
L Inferior frontal gyrus (BA 9)	−50	20	20	3.29	87
L Angular gyrus (BA 39)	−52	−62	30	3.30	73
**Negative correlation**					
L Anterior cingulate (BA 33)	−2	18	18	3.71	89
R Cingulate gyrus (BA 32)	14	16	26	3.16	
L Cingulate Gyrus (BA 24)	−6	18	26	2.71	
R Caudate tail	30	−38	6	3.69	178
R Parahippocampal gyrus (BA 19)	40	−44	−6	3.29	
R Middle temporal gyrus (BA 21)	48	0	−16	3.43	141
R Superior temporal gyrus (BA 22)	48	−2	−4	3.10	
L Middle occipital gyrus (BA 19)	−46	−82	18	3.41	94
**ERROR TRIALS**					
**Positive correlation**					
L Parahippocampal gyrus (BA 28)	−18	−18	−26	4.16	83
**Negative correlation**					
R Medial frontal gyrus (BA 6)	14	−24	58	3.80	74
R Superior frontal gyrus (BA 8)	12	44	46	3.24	93
	16	48	38	2.63	
	0	48	46	2.63	
L Superior temporal gyrus (BA 39)	−48	−56	24	3.44	105
L Inferior parietal lobule (BA 40)	−52	−58	36	2.80	
R Inferior parietal lobule (BA 40)	54	−52	44	2.91	72
R Angular gyrus (BA 40)	56	−56	36	2.79	

Brain regions showing significant positive and negative correlations across the total sample (N = 24) on successful and erroneous perceptual conflict trials (p<.005 uncorr., k>70 voxels).

On successful trials (Figure 4A; Table 5) increased performance-related activity was seen in inferior frontal gyrus (IFG; BA 9) and angular gyrus (ANG; BA 39) (upper panel). A negative association (lower panel) was evident in limbic (BA's 19, 24, 32, 33), temporal (BA's 21, 22) and occipital brain regions (BA 19) as well as in the caudate tail.

On error trials (Figure 4D; Table 5) increase in performance was positively correlated with activation in the parahippocampal gyrus (PHG; BA 28) only (upper panel). Furthermore, with increasing performance also activation decreased in frontal (BA's 6, 8) and temporal (BA 39) brain regions (lower panel).

ROI analyses were performed for all brain regions that have been displayed by regression analyses across successful and error trials.

Across successful trials only activity in the left ANG (BA 39) yielded significance between good and poor performers (t_(14)_ = −3.912, p = .022) (Figure 4B). Although other brain regions did not survive the statistical threshold set, additional regression analysis (Figure 4C) revealed strong relationships between performance score and beta estimates. A positive correlation with task performance, indicating that decreasing performance was associated with higher beta estimates, was evident in left ANG (BA 39) (r = .505, p = .012). Negative correlations were evident in left (r = .600, p = .002) and right (r = .414, p = .044) anterior cingulate (ACC; BA 33), left cingulate gyrus (CG; BA 24) (r = .461, p = .023), right middle temporal gyurs (MTG; BA 21) (r = .551, p = .005) and right superior temporal gyrus (STG; BA 22) (r = .538, p = .007), implying that decreasing performance was associated with lower beta estimates in these regions.

Across error trials differences between good and poor performers were not significantly different in any region (Figure 4E). However, as indicated by regression analyses (Figure 4F) task performance was negatively correlated with beta estimates in left PHG (BA 28) (r = .448, p = .028) and positively correlated in the right medial frontal gyrus (MeFG; BA 6) (r = .411, p = .046).

#### Distinct recruitment of neural networks by good and poor performers

**Figure 5:**
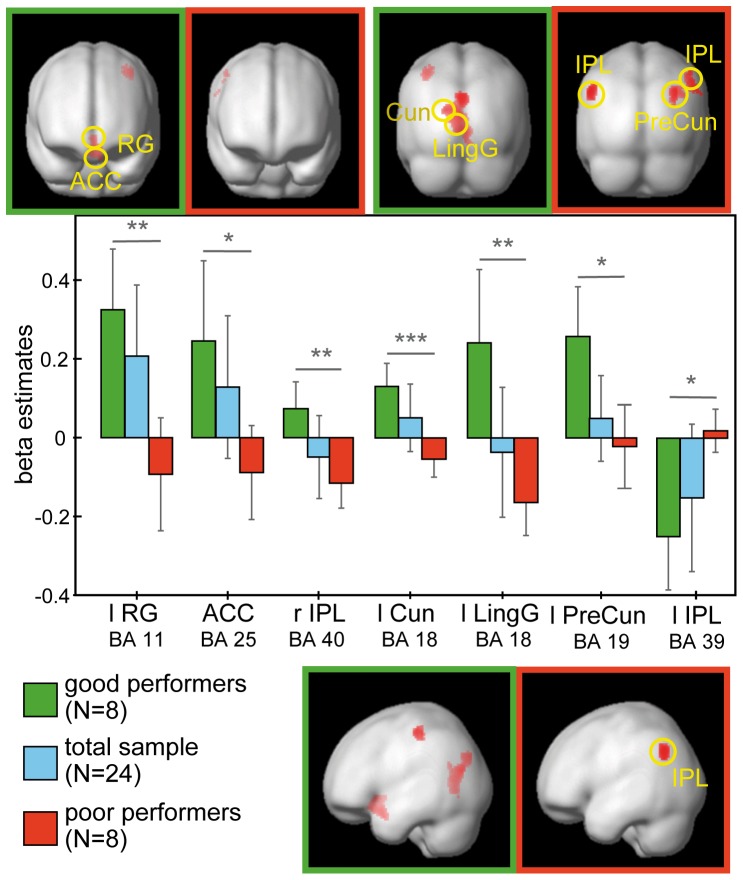
Differential neuronal responses of performance during perceptual conflict. Regions of activation associated with the perceptual conflict condition between good and poor performers (p<.005 uncorr., k>70 voxels) and corresponding beta estimates. Using ANOVAs activation clusters elicited by good performers were seen in the RG (BA 11), ACC (BA 25), IPL (BA 40) and visual areas V2 and V3. Poor performers robustly activated only the IPL (BA 39). Corresponding beta estimates revealed significant differences between good and poor performers in any of these regions.

Figure 5 illustrates the activation clusters obtained by good and poor performers of the extreme groups. The corresponding voxel locations and statistical information are listed in Table 6.

**Table 6:** Activation patterns in good and poor performers on perceptual conflict trials.

Region	Talairach coordinates			Z	k_E_
	x	y	z		
**Main effect of Performance**					
L Rectal gyrus (BA 11)	−4	10	−22	3.32	96
	−6	18	−20	2.94	
L Anterior cingulate (BA 25)	0	14	−8	2.78	
L Cuneus (BA 18)	−18	−74	18	3.93	301
L Lingual gyrus (BA 18)	−4	−66	2	3.55	
R Culmen of Vermis	6	−62	−2	3.58	
L Cuneus (BA 18)	0	−80	28	3.36	76
L Cuneus (BA 7)	−6	−74	30	2.82	
**Good>poor performers**					
L Postcentral gyrus (BA 3)	−38	−28	50	3.26	90
L Precentral gyrus (BA 4)	−32	−30	56	2.83	
L Rectal gyrus (BA 11)	−4	10	−22	3.51	216
L Anterior cingulate (BA 25)	0	14	−8	3.00	
L Cuneus (BA 18)	−18	−74	18	4.09	654
L Lingual gyrus (BA 18)	−4	−66	2	3.72	
R Culmen of Vermis	6	−62	−2	3.75	
**Poor>good performers**					
R Middle temporal gyrus (BA 39)	32	−60	28	3.59	159
R Inferior parietal lobule (BA 40)	38	−50	34	3.20	
R Precuneus (BA 19)	34	−66	36	2.99	
L Inferior parietal lobule (BA 39)	−50	−62	38	3.57	92
R Inferior parietal lobule (BA 40)	50	−50	44	2.95	82
	54	−50	36	2.85	
R Supramarginal gyrus (BA 40)	62	−48	32	2.62	

Brain regions significantly activated across good (N = 8) and poor performers (N = 8) on perceptual conflict trials as derived from the ANOVA (p<.005 uncorr., k>70 voxels).

On successful perceptual conflict (LOB) trials the main effect of performance yielded significant activation in fronto-limbic (BA's 11, 25) and occipital (BA's 7, 18) brain areas. Pair-wise comparisons revealed that this effect was mainly driven by the contrast of good>poor performers yielding prominent activation clusters in frontal (BA's 4, 11), limbic (BA 25), parietal (BA 3) and visual association areas (BA 18) and in the cerebellar culmen of vermis for good performers.

The reversed contrast of poor>good performers revealed a stronger activation cluster for poor performers only in temporo-parietal brain regions encompassing BA's 19, 39 and 40.

On error trials the main effect of performance yielded statistical significance neither at the same threshold nor at a lower threshold of 10 voxels uncorrected at a level of p<.001. Therefore, performance-related differences in the recruitment of neural networks seem to be based on different strategies developed for successful completion of the task at hand.

ROI analyses were conducted on the basis of the ANOVA across correct trials, yielding significant differences in beta estimates exclusively in left-lateralized fronto-parietal and occipital regions. Performance differences between good and poor performers were found to be significant in the rectal gyrus (RG; BA 11) (t_(14)_ = 4.688, p = .005), inferior parietal lobule (IPL; BA's 39, 40) (all t_(14)_<4.788, p<.026), cuneus (Cun) and lingual gyrus (LingG; BA 18) (all t_(14)_<5.860, p<.005) and marginally in the ACC (BA 25) (t_(14)_ = 3.348, p = .094). One sample t-tests revealed significant activation for good performers only left-lateralized in the RG (BA 11) (t_(7)_ = 4.971, p  = .024), Cun (BA 18) (t_(7)_ = 5.271, p = .017) and the precuneus (PreCun; BA 19) (t_(7)_ = 4.829, p = .029). Significant deactivations for good performers were evident only in IPL (BA 39) (t_(7)_ = −4.359, p = .05). In contrast, for poor performers the LingG (BA 18) yielded significant deactivation (t_(7)_ = −4.625, p = .036) as well as the left IPL (BA 40) (t_(7)_ = −4.288, p = .054).

## Discussion

In the current study we investigated neural correlates of perceptual conflict that determine performance in competitive attentional selection. Participants were instructed to detect changes in luminance (task-relevant stimulus) accompanied by contralateral changes in orientation (task-irrelevant stimulus), inducing a perceptual conflict [29]. Our results support theories that claim an interaction of bottom-up and top-down processes mediated by distinct attentional networks [14], [17], [38]–[41].

Both, our behavioral and fMRI data indicated that detection of the task-relevant luminance change was influenced by the simultaneously presented distractor of orientation change if both were spatially separated (see also [29]). The behavioral data yielded faster reaction times on non-conflict trials of LUM and LOU (for the ORI condition no response was required) and higher accuracy in all non-conflict conditions of LUM, ORI and LOU as compared to the perceptual conflict trials (LOB) with good performers outperforming the poor performers concerning error rates (Table 2). Accordingly, the fMRI data indicated that interindividual differences are accompanied by distinct activation patterns during successful performance on a competitive attentional task (Figure 5). The results of the random effects analyses, ANOVA and beta estimates across the performance groups presented areas that are activated to varying degrees by good and poor performers. Good performers showed strong neuronal enhancement of fronto-parietal and visual brain areas and a decrease in brain activation in parietal areas involving BA 39. In contrast, poor performers depicted a reversed activation pattern, showing neuronal enhancement only in BA 39 and neuronal suppression in fronto-parietal and visual brain areas whereby the modulation of neuronal enhancement and suppression was not as strong as it has been shown by the good performers. Together, these results indicate a distinct recruitment of fronto-parietal and visual areas that contribute differentially to varying degrees of visual processing capacities and top-down control.

Top-down attentional processes have repeatedly been hypothesized to recruit a network of fronto-parietal areas [5], [17]–[18], [42], in which prefrontal areas and the anterior cingulate cortex (ACC) may provide a source of top-down attentional control [26], [43]–[47]. Our results revealed a strong engagement of the orbitofrontal cortex (BA 11), cingulate and anterior cingulate cortex in good performers which is in accordance with this assumption. The orbitofrontal cortex (OFC) has dense reciprocal connections with prefrontal, limbic and sensory areas and is most heavily linked to the medial and inferior temporal lobe [48]–[52], areas known to be part of the ventral processing stream [53]–[54]. Similarly, the ACC integrates incoming inputs and has strong reciprocal interconnections with lateral prefrontal, parietal cortex, premotor and supplementary motor areas [55]. Therefore, increased activity in OFC and ACC obtained by good performers relative to poor performers may reflect differences in recruiting top-down attentional control. Moreover, the OFC and ACC act upon brain regions that modulate sensory processing [56]–[57] which may be reflected in our activation pattern found in extrastriate visual areas (BA's 18, 19). Thus, it can be assumed that increased brain activation in OFC and ACC revealed by good performers reflect a stronger recruitment of top-down attentional control to support processing of the task-relevant stimulus [58]–[59]. In line with this interpretation, poor performers showed less recruitment of the OFC and ACC as indicated by decreased beta estimates. Decreased brain activation in poor performers might reflect less focused attention and thus weaker top-down attentional control to resolve the perceptual conflict.

Besides the involvement of OFC and ACC in top-down control and sensory modulation, alternative hypotheses relate OFC and ACC function to reward [60]–[63], effort computation [61], [64], and performance monitoring [65]. Although we did not offer reward for good task performance some participants may have encouraged and maintained task compliance in a way that they experienced reward even only by complying with the task instruction or felt a rewarding effect of success. This would be in accordance with the incentive salience hypothesis, which combines both perceptual and motivational features by attributing motivational values to neutral stimuli that previously carried purely perceptual information [66]–[67]. In this sense, the attribution of incentive salience to the task-relevant change of luminance could have led to differential recruitment of the OFC.

Another approach that results in different performance outcomes involves models of effort computation and decision making. Decision making implies a selection of an option over another on the basis of preferences and the balance between costs and benefits associated with pursuing one option [68]. As subjects experience cost-benefit decision differently, some may exert higher effort to obtain a desired option than other subjects. Neuroimaging studies have shown that the ACC evaluates the benefit of an option by integrating both the expected outcome and the effort needed [61], [67]–[70]. Therefore, differences in ACC activation may reflect different levels of effort invested by good and poor performers. Together, these hypotheses provide an insight into orbitofrontal and ACC function that could be the consequence rather than the cause of successful trials.

In line with the modulation of activity found in frontal areas, the results pattern in the inferior parietal lobule (BA 40) similarly revealed an increase in activation for good performers and a decrease in activation for poor performers. As proposed by Posner and Petersen [40] the posterior attention system operates upon the ventral stream which is required for detailed object identification. Accordingly, this model has been confirmed by previous research implicating the inferior parietal cortex in top-down attentional control [57], [71], especially in target detection [72]–[73].

In addition to fronto-parietal projection sites of top-down attentional control, good performers revealed higher activation in visual areas, compared to poor performers. Recent work has shown that neural mechanisms of visual selective attention operate at cortical and subcortical stages in the visual system [8], [15], [19], [74] and also the biased competition account assumes that perceptual conflicts are resolved in visual cortical areas [3], [5]. The observed increased brain activity in areas V2 and V3 in good performers and decreased activity in these areas for poor performers is in line with the above findings and may additionally contribute to overt performance differences. As revealed by one-sample t-tests, beta estimates for good performers in V2 and V3 increased significantly whereas beta estimates for poor performers decreased and showed only significant activity in the left lingual gyrus. This is in line with the findings from fronto-limbic areas indicating that increasing activation in prefrontal areas and the ACC provide stronger top-down attentional control that exert effects on visual areas to resolve perceptual conflict.

In contrast to increasing activation in visual areas V2 and V3, good performers presented significant deactivation in BA 39 that corresponds to the monkey inferotemporal cortex, an area known to be involved in high-level visual processing, especially in internal representation and target selection [11]. Moreover, the angular gyrus has been suggested to house a salience map [17], [47] with a winner-take-all process selecting the most salient stimulus from the current visual set and shifting attention towards this location [75]–[77]. In this sense, a strong deactivation as reflected by good performers may indicate an active suppression of the task-irrelevant change of orientation which has been accounted as the most salient. Poor performers did not reveal such a deactivation of BA 39. It is therefore conceivable that deactivation of BA 39 is part of a ‘strategy’ utilized by good performers to reduce processing of the salient, but irrelevant orientation change, which causes the competition with the less salient, but behaviorally relevant luminance change.

In summary, the results show a correlation between brain activation and individual performance differences in competitive attentional selection. Good performance on the task was reflected in higher accuracy and increased activation of fronto-parietal and visual areas V2 and V3, but also in decreased activation in BA 39. The latter deactivation may be part of a ‘strategy’ to reduce influences of the salient distractor in the bottom-up channel. On a related note, deactivations in areas that have been related to top-down attentional control were closely tied to poor performance that may indicate deficient top-down attentional control. These results confirm previous studies emphasizing the importance of a fronto-parietal network that has been shown to be necessary to exert top-down attention control. With our study we extended the available expertise by highlighting the varying recruitment of the fronto-parietal network during situations of perceptual conflict.
